# Temperature-associated microbial succession and volatile flavor compound dynamics during cigar tobacco leaf fermentation

**DOI:** 10.1186/s40643-026-01060-1

**Published:** 2026-06-04

**Authors:** Tianfei Zheng, Dongfeng Guo, Yaqi Shi, Jinlong Zhou, Cunyong Zhang, Kun Zong, Shaoxuan Ju, Xingjiang Li

**Affiliations:** 1 School of Food and Biological Engineering, Hefei University of Technology, Hefei, 230009 China; 2 Technology Center, China Tobacco Anhui Industrial Co. Ltd., Hefei, 230088 China

**Keywords:** Cigar tobacco leaves, Fermentation temperature, Microbial network, Community succession, Volatile flavor compounds

## Abstract

Temperature is a key factor driving microbial community succession and volatile flavor compound formation during the fermentation of cigar tobacco leaves (CTLs). This study systematically investigated microbial community dynamics, co-occurrence networks, and volatile flavor compound (VFCs) profiles of CTLs from Dominica and Yunnan under a 20–60 °C fermentation gradient. High-throughput sequencing identified *Staphylococcus*, *Oceanobacillus*, and thermophilic fungi as core microbes potentially associated with aroma formation. Dominica CTLs exhibiting higher microbial diversity than Yunnan CTLs. Dominica CTLs produced abundant esters, alcohols and ketones across different temperature stages, whereas Yunnan CTLs accumulated more pyrazines, indole and terpenoids at high temperatures (≥ 50 °C). Co-occurrence network analysis revealed temperature-driven shifts in microbial interactions: Dominica CTLs formed balanced networks with mixed positive/negative correlations at low temperatures, while Yunnan CTLs developed stable networks dominated by positive correlations at high temperatures. PERMANOVA indicated significant differences in microbial community structure among temperature gradients (R^2^ = 0.78, *p* < 0.001). Spearman correlation analysis suggested that core microbes (e.g., *Staphylococcus*) were significantly correlated with the accumulation of key VFCs (e.g., esters, alcohols). These findings propose a conceptual temperature–microbe–VFC interaction framework, providing theoretical support for optimizing CTL fermentation processes.

**Graphical abstract:**

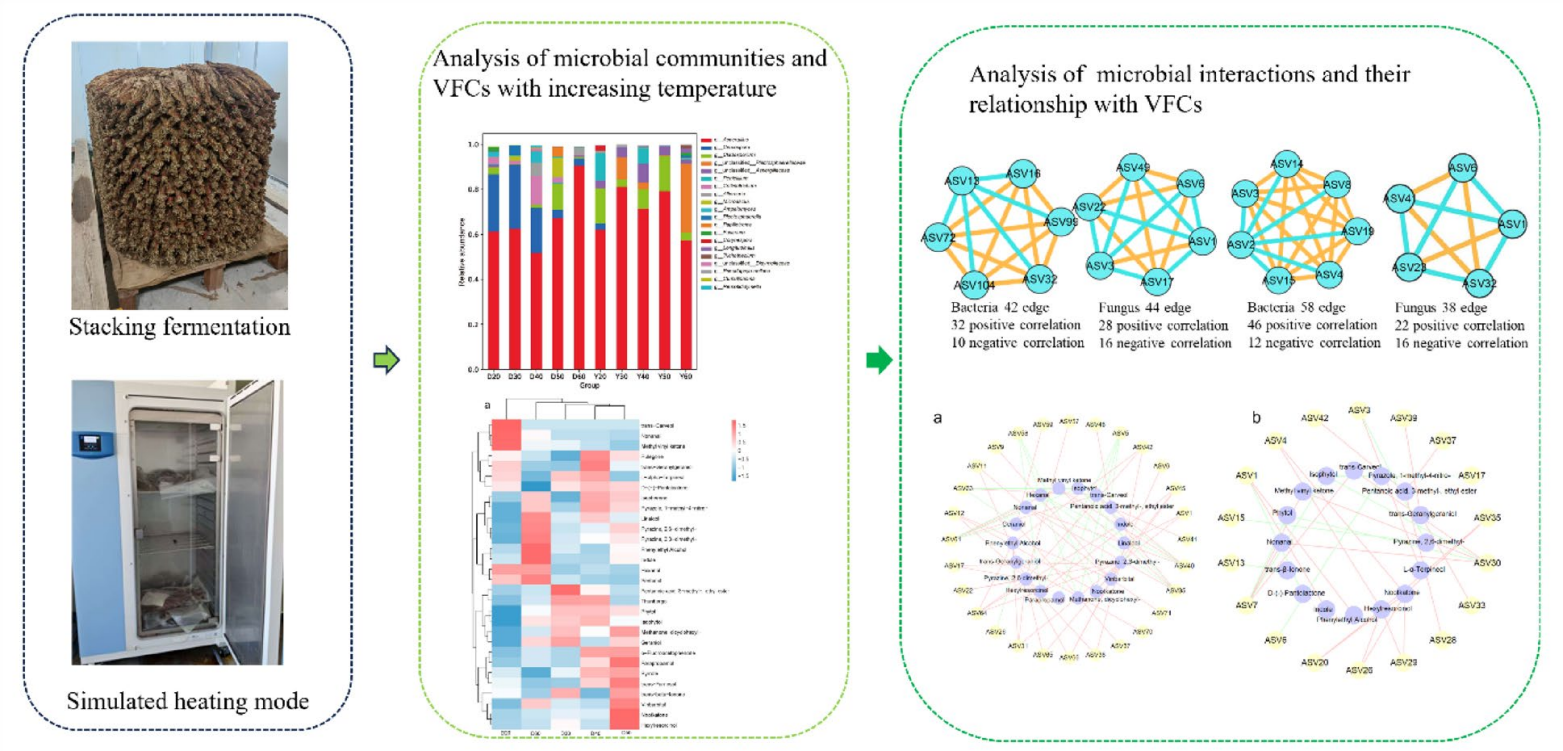

## Introduction

The fermentation of cigar tobacco leaves (CTLs) is a typical solid-state fermentation process dominated by microorganisms (Ma et al. 2024; Yu et al. 2025). Microbial communities involved in fermentation include bacteria (e.g., *Bacillus*, *Pseudomonas*), actinomycetes (e.g., *Streptomyces*), and fungi (e.g., *Aspergillus*, *Saccharomyces*) (Zheng et al. 2022). These microorganisms secrete hydrolytic enzymes such as cellulase, protease, and amylase to degrade substrates in CTLs (Zhang et al. 2023b), and produce flavor compounds like organic acids, esters, and ketones (Wu et al. 2023, 2025). For instance, *Bacillus* can degrade nicotine, reducing the bitter and astringent taste (Shan et al. 2025; Wei et al. 2024); certain fungi (e.g., *Aspergillus oryzae*) can synthesize furan compounds, contributing caramel-like aromas (Wieder et al. 2025). The succession and functional differentiation of microbial communities directly determine fermentation efficiency and product quality (Zhang et al. 2025; Cai et al. 2025). Temperature is a core regulatory driver of CTL fermentation. Traditional natural stacking fermentation is characterized by dynamic temperature changes driven by microbial metabolic heat (Zhang et al. 2023a). During industrial stack fermentation, stack temperature increases by 2–3 °C daily, shifting from low (30–40 °C) to high (50–60 °C) ranges, forming an alternating dominance of mesophilic and thermophilic microorganisms (Zhang et al. 2024a, b). Low-temperature stages (< 40 °C) favor mesophilic microbes (*Pseudomonas*, *Saccharomyces*), whose metabolic heat further raises stack temperature; when temperature exceeds 45 °C, heat-resistant bacteria become dominant and mesophilic microbial growth is inhibited. Temperature fluctuations directly regulate microbial metabolic activity, and indirectly shape microbial community structure by altering enzyme activity (Song et al. 2024), membrane fluidity (Knapp et al. 2025), and interspecific competitive relationships (Han et al. 2025), thereby affecting fermentation product quality.

Existing studies have investigated the impact of temperature on the microbial community in CTLs. Ren et al. (2023) found that increasing fermentation chamber temperature above 27 °C enhanced microbial diversity and aroma quality. Jia et al. (2025) reported that *Staphylococcus*, *Stemphylium*, *Sampaiozyma*, and *Filobasidium* were biomarkers in the low-temperature fermentation group, associated with flavor metabolite production, while *Aspergillus*, *Neodymella*, *Acinetobacter*, *Pelomonas*, *Brevundimonas*, and *Alkalihalobacillus* dominated medium- and high-temperature groups, potentially contributing to nitrogen compound degradation. However, most studies focus on single temperature points and lack systematic analysis of microbial dynamics under a continuous temperature gradient. Moreover, integration of microbiome and metabolome data across different CTL origins remains limited.

This study targeted CTLs from Dominica (Corojo) and Yunnan (Yunxue 1), simulating industrial temperature rise patterns. Using high-throughput sequencing and HS–SPME–GC–MS, we analyzed microbial community succession and VFC dynamics across temperature gradients. Correlation analyses and network construction were employed to reveal potential associations between microbial communities and VFC formation. This work provides insights into temperature-associated microbial and volatile dynamics, offering a theoretical basis for optimizing CTL fermentation processes.

## Materials and methods

### Fermentation and sample collection of cigar tobacco leaves

CTLs from Dominica (15° N, 61° W) and Yunnan (Yuxi, 24° N, 102° E, China) were selected. Leaves were stored at 20 °C, 60% RH for 7 d before fermentation, then rehydrated to a moisture content of 30 ± 0.5% (consistent with industrial practice (Gao et al. 2025)). Fermentation was performed in a temperature- and humidity-controlled incubator (85 ± 2% RH) using 5 L breathable polypropylene boxes with 0.22 μm membrane vents (oxygen concentration 18–21%).

Initial fermentation temperature was 20 °C, increased by 2.5 °C daily (total 16 d), consistent with industrial stack fermentation dynamics (Jia et al. 2025; Zhang et al. 2024a, b). Temperature uniformity was verified by multi-point recording (≤ ± 0.5 °C deviation). Samples were collected at 20 °C, 30 °C, 40 °C, 50 °C, and 60 °C, with three biological replicates per temperature point. Moisture content (28–32%) and weight loss (0.3 ± 0.05% d⁻^1^) were monitored. Samples were frozen in liquid nitrogen and stored at − 80 °C. Dominica CTLs were labeled D20-D60, Yunnan CTLs as Y20-Y60.

### Analysis of volatile flavor compounds in cigar tobacco leaves

VFCs in CTLs were detected by headspace solid phase microextraction-gas chromatography-mass spectrometry (HS–SPME–GC–MS) referring to the method of Kind et al. with minor modifications (Kind et al. 2009). CTLs were ground into powder after being frozen in liquid nitrogen and then loaded into a 20 mL headspace bottle. 10 μL of 2-octanol (10 mg/L stock in dH_2_O) was added as an internal standard semi-quantitative analysis. The volatile compounds in CTLs were extracted by headspace solid-phase microextraction (50/30 μm DVB/CAR/PDMS fibre, Supelco, Bellefonte, PA, USA) at 60 °C for 30 min. After extraction, the volatile compounds were identified using a Pegasus BT GC-TOFMS (LECO Co., St. Joseph, MI, USA), with a DB-Wax column (30 m × 250 μm × 0.25 μm). Helium C-60 was used as a carrier gas with a flow rate of 1 mL/min, and the injector port was heated to 250 °C. The oven temperature was initially fixed at 40 °C for 2 min, then increased to 250 °C at a rate of 10 °C/min and held for 5 min. The injection, transfer line, ion source and quad temperatures were 250, 250, 230 and 150 °C, respectively. The energy was -70 eV in electron impact mode. The mass spectrometry data were acquired in scan mode with a m/z range of 20–400 and a solvent delay of 2.37 min.

Chroma TOF 4.3X software (LECO Corporation) and the Nist database were used for raw peak processing and compound identification. The similarity threshold for NIST database matching was ≥ 80%; retention index calibration was performed using C8-C40 n-alkanes with a retention index matching degree ≥ 90%; response factor correction was applied for esters, alcohols and ketones separately. Each sample was analyzed with 3 technical replicates, and results were averaged. Key compounds (nootkatone, indole, 2,3-dimethyl-pyrazine) were confirmed by authentic standards. The top 30 VFCs by relative abundance (cumulatively accounting for > 85% of total VFCs) were selected for subsequent analysis.

### Analysis of microbial communities in cigar tobacco leaves

Amplicon sequencing was used to analyze the impact of temperature rise on CTL microbial communities. The microbial communities in CTLs were collected by washing with normal saline. Briefly, 5 g of CTLs were homogenized in 100 mL of sterile normal saline and agitated at 220 rpm for 4 h to dislodge adherent microorganisms. The suspension was sequentially filtered through sterile gauze and centrifuged at 7000 × g for 15 min to collect microbes, which were stored at − 20 °C until processing. Total genomic DNA of the microbial communities in CTLs were extracted using the DNeasy PowerSoil Kit (QIAGEN) according to the manufacturer's protocol. The quality and concentration of the extracted DNA were assessed using a NanoDrop ND-1000 spectrophotometer (Thermo Fisher Scientific) and agarose gel electrophoresis.

The V3-V4 region of bacterial and archaeal 16S rRNA genes was amplified with forward primer 341F (5′-ACTCCTACGGGAGGCAGCAG-3′) and reverse primer 806R (5′-GGACTACHVGGGTWTCTAAT-3′). The fungal internal transcribed spacer (ITS) gene was amplified with universal primers ITS1F (5′-CTTGGTCATTTAGAGGAAGTAA-3′) and ITS2R (5′-GCTGCGTTCTTCATCGATGC-3′). Amplified PCR products were purified and quantified, then sequenced on an Illumina NovaSeq PE250 platform (2 × 300 bp chemistry) following standard protocols. A sterile normal saline blank control and a PCR blank control were set up, with an average sequencing depth of < 500 reads in blank controls and no obvious microbial signals.

Raw reads were processed using DADA2 for primer removal, quality control, denoising, splicing, and chimera removal to obtain amplicon sequence variants (ASVs) (Callahan et al. 2016); the decontam R package was used to remove contaminant sequences detected in blank controls. Obtained ASVs were annotated and analyzed using QIIME2 (Bolyen et al. 2019), with bacterial annotation based on the SILVA 138 database and fungal annotation on the UNITE 8.3 database. The average sequencing depth was 45,600 ± 3200 reads for bacterial 16S rRNA and 38,900 ± 2800 reads for fungal ITS per sample; rarefaction was performed at a depth of 30,000 reads per sample based on rarefaction curves to ensure uniform sequencing depth.

### Statistical analysis

All statistical analyses were performed using R v.4.0.0. Shapiro–Wilk normality test and Levene variance homogeneity test were used to verify ANOVA assumptions; indices that did not meet the assumptions were analyzed by Kruskal–Wallis nonparametric test. Differences in alpha diversity indices and relative abundances of dominant microbes/VFCs among temperature groups were tested by one-way ANOVA followed by Tukey's multiple comparison test (*p* < 0.05). Microbial relative abundance data were subjected to centered log-ratio (CLR) transformation before statistical analysis to reduce compositional data bias.

Heatmaps of dominant VFCs were generated using the heatmap package in R; Venn diagrams were constructed using the Venn Diagram package to analyze shared and unique ASVs among different temperatures. Linear discriminant analysis effect size (LEfSe) was used to identify differentially abundant microbial taxa (biomarkers) with LDA score > 4.0 and Bonferroni-corrected *p* < 0.05. Beta diversity was analyzed by principal coordinate analysis (PCoA) based on Bray–Curtis distance, and PERMANOVA was used to verify significant differences in microbial community structure (*p* < 0.05).

Spearman's correlation coefficient was used for correlation analysis between top 30 ASVs (by abundance) and top 30 key VFCs (by abundance), with Benjamini–Hochberg correction for multiple testing (FDR < 0.05). PCoA was used to analyze VFC differences among temperature gradients and producing areas. Co-occurrence networks were constructed using Gephi 0.9.2 software with correlation coefficients |r|> 0.8 and *p* < 0.05. The microbial co-occurrence networks topological indices (average degree, modularity, network density) were calculated as undirected multigraphs, where multiple edges between the same pair of nodes were permitted to preserve all significant correlations (both positive and negative) from different temperature stages or analytical thresholds.

## Results and discussion

### Dynamic succession of microbial communities with increasing temperature

**Fig. 1:**
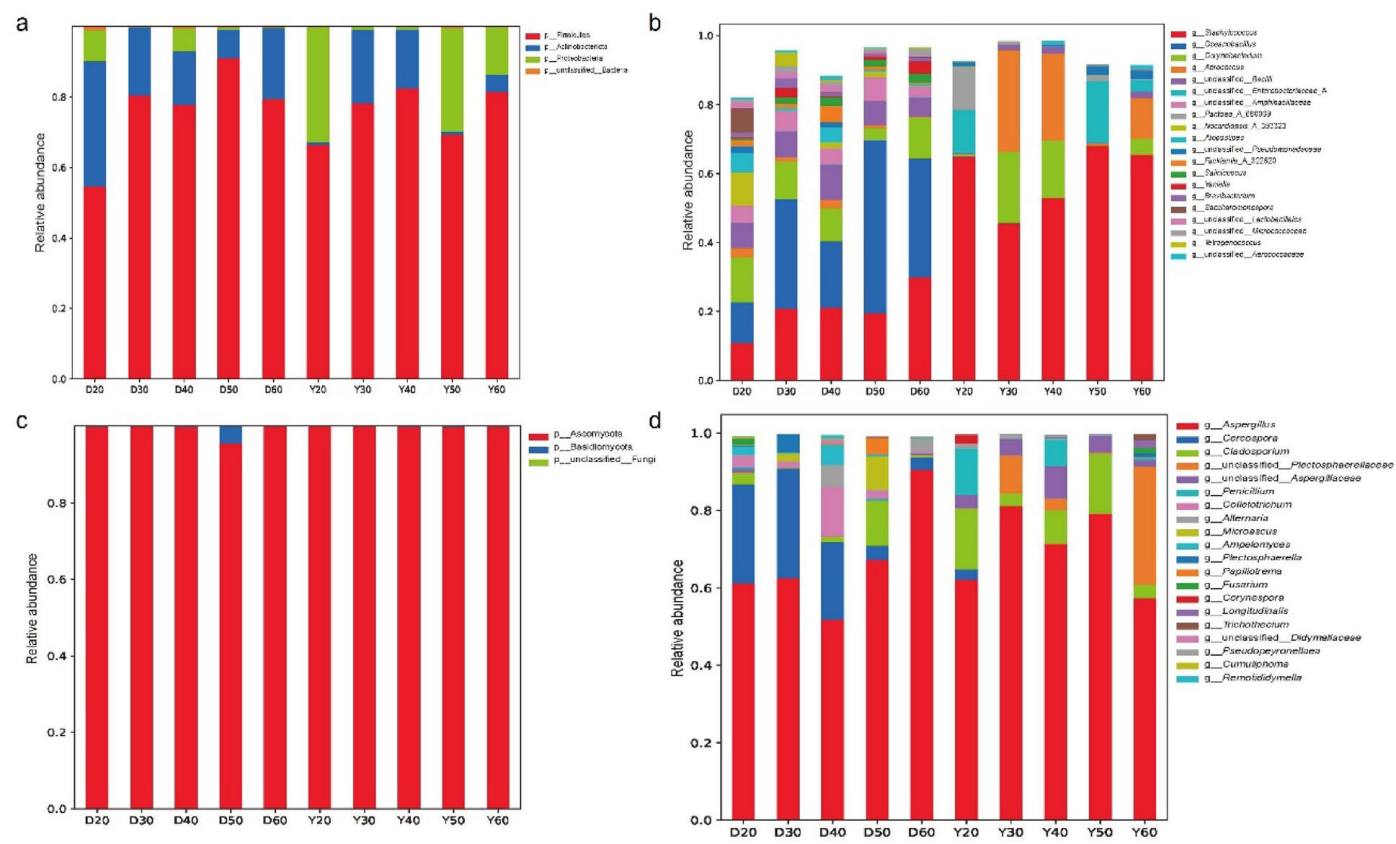
Composition of microbial communities in CTLs from Dominica and Yunnan at different fermentation temperatures. **a** Dominant bacterial phyla in Dominica CTLs; **b** dominant bacterial genera in Dominica CTLs; **c** dominant fungal phyla in Yunnan CTLs; **d** dominant fungal genera in Yunnan CTLs

Microbial community composition in CTLs from both origins exhibited distinct temperature-driven patterns (Fig. 1). At the phylum level, Firmicutes, Actinobacteriota, and Proteobacteria dominated bacterial communities (> 85% of total abundance). Firmicutes, the most abundant phylum, is known for producing hydrolytic enzymes (cellulase, protease) during tobacco fermentation (Zhang et al. 2025). Dominant fungal phyla were Ascomycota and Basidiomycota, consistent with previous studies (Wu et al. 2023).

At the genus level, origin-specific community structures were evident. Dominica CTLs harbored relatively even abundances of *Staphylococcus*, *Oceanobacillus*, *Corynebacterium*, *Aspergillus*, and *Cercospora*. In contrast, Yunnan CTLs were dominated by *Staphylococcus* (mean relative abundance 59.3%), *Aerococcus*, and *Corynebacterium*. *Oceanobacillus* was characteristic of Dominica CTLs (mean 29.5%) but scarce in Yunnan CTLs (0.5%). These differences likely reflect initial microbial colonization shaped by climate and soil conditions (Zheng et al. 2022).

With increasing fermentation temperature, the relative abundance of dominant genera changed dynamically. In Dominica CTLs, *Staphylococcus*, *Oceanobacillus*, *Corynebacterium*, and *Cercospora* increased with temperature, while *Aspergillus* decreased. In Yunnan CTLs, *Staphylococcus* abundance peaked at 60 °C (65.4%), consistent with its thermotolerant traits (Nazipi et al. 2021; Yang et al. 2024). *Corynebacterium* and *Aspergillus* peaked at 40 °C, reflecting distinct temperature adaptability ranges. The ecological succession from mesophilic to thermophilic microbial communities directly shapes the functional output of CTL fermentation. Mesophilic genera such as *Corynebacterium* and *Cercospora* (dominant at 20–40 °C) are known producers of extracellular hydrolases (e.g., proteases, pectinases) that break down tobacco leaf macromolecules, releasing fermentable sugars and amino acids (Zhang et al. 2024a, b). These precursors subsequently fuel the biosynthesis of esters and higher alcohols, which are key contributors to fruity and floral notes in the final product. Conversely, thermophilic taxa including *Oceanobacillus* and *Staphylococcus* (enriched at 50–60 °C) exhibit high-temperature metabolic activity, including the Maillard reaction-promoting amino acid decarboxylation and terpene cyclization pathways. The accumulation of pyrazines, indole, and nootkatone at high temperatures ("Changes in volatile flavor compounds with increasing temperature" section) is consistent with the metabolic potential of these thermophilic communities. Thus, the temperature-driven transition represents a functional handover: mesophilic stages prime the substrate for flavor generation, while thermophilic stages drive the formation of characteristic heat-induced aroma compounds.

The temperature-driven restructuring of microbial communities involves multiple mechanisms. Enzymatic activity favors thermophiles above 50 °C as mesophilic enzymes denature (Vala et al. 2025). Membrane adaptation enables thermophiles like *Staphylococcus* and *Oceanobacillus* to maintain integrity via saturated fatty acid enrichment (Nazipi et al. 2021). Metabolic heat creates a positive feedback loop: mesophilic activity raises temperature, suppressing their own growth while selecting for thermophiles (Zhang et al. 2024a, b). Temperature also alters competitive dynamics, as evidenced by *Aspergillus* decline coinciding with heat-tolerant bacterial proliferation. Together, these mechanisms explain the observed successional patterns.

**Fig. 2:**
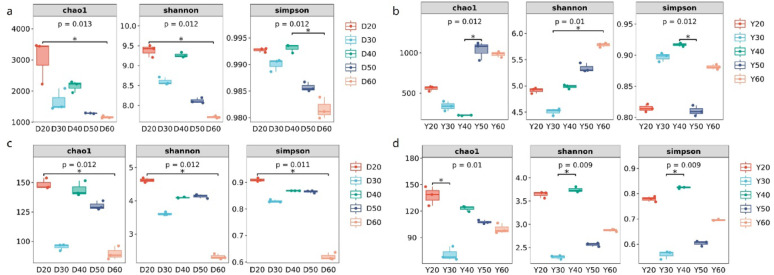
Alpha diversity of microbial communities in CTLs from Dominica and Yunnan at different fermentation temperatures. **a** Bacterial communities in Dominica CTLs; **b** bacterial communities in Yunnan CTLs; **c** fungal communities in Dominica CTLs; **d** fungal communities in Yunnan CTLs

Alpha diversity indices (Chao1, Shannon, Simpson) were used to evaluate microbial community richness and evenness (Han et al. 2026). Different letters in (a-d, Fig. 2) indicated significant differences among temperature groups (*p* < 0.05, Tukey's test). In Dominica CTLs, Chao1 (richness) and Shannon (evenness) indices decreased continuously with temperature. This decline suggests that high temperatures reduce community complexity and may narrow functional breadth, potentially focusing metabolic activity on heat-stable aroma formation. In Yunnan CTLs, bacterial diversity first decreased (20–40 °C) then increased (40–60 °C), likely due to enrichment of unique thermophilic ASVs (30.3% at 50 °C, 26.4% at 60 °C). This biphasic pattern indicates that while initial temperature increases reduce diversity, further warming promotes a specialized thermophilic community that maintains functional capacity under thermal stress. Fungal diversity decreased continuously in both origins, suggesting fungi are generally more temperature-sensitive than bacteria.

**Fig. 3:**
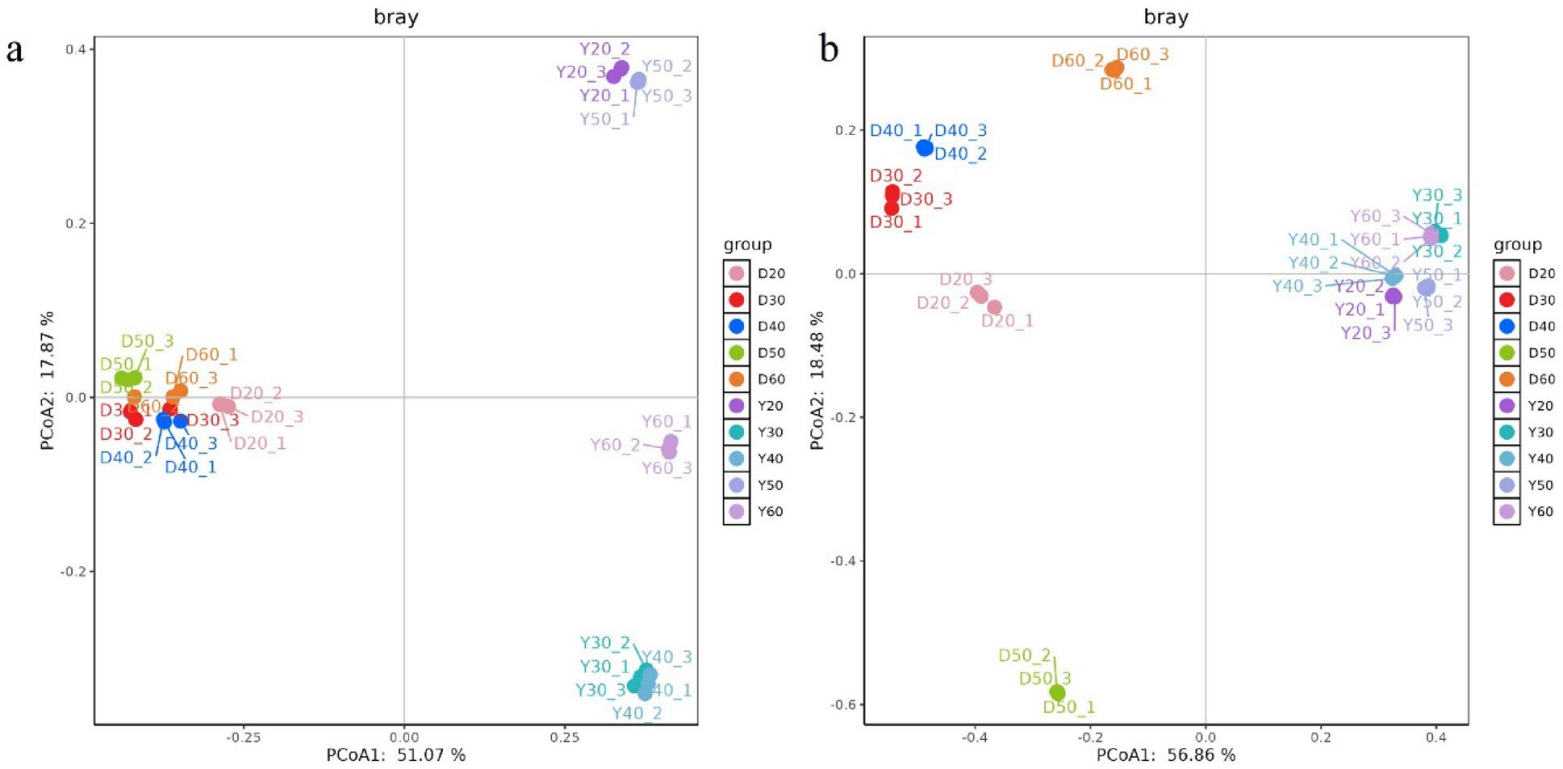
Beta diversity of microbial communities in CTLs from Dominica and Yunnan at different temperatures. **a** PCA of bacterial communities based on Bray–Curtis distance; **b** PCA of fungal communities based on Bray–Curtis distance

Beta diversity analysis (PCoA, Fig. 3) showed clear separation of microbial communities across temperatures. PERMANOVA confirmed significant differences (bacteria: R^2^ = 0.75, *p* < 0.001; fungi: R^2^ = 0.82, *p* < 0.001). For Dominica CTLs, bacterial communities clustered tightly within each temperature group but still differed significantly across temperatures, indicating subtle but functionally meaningful restructuring rather than complete stability. Fungal communities showed clear separation, with 50 °C as a critical threshold. For Yunnan CTLs, bacterial communities were more scattered, reflecting stronger temperature sensitivity, while fungal communities remained relatively stable.

**Fig. 4:**
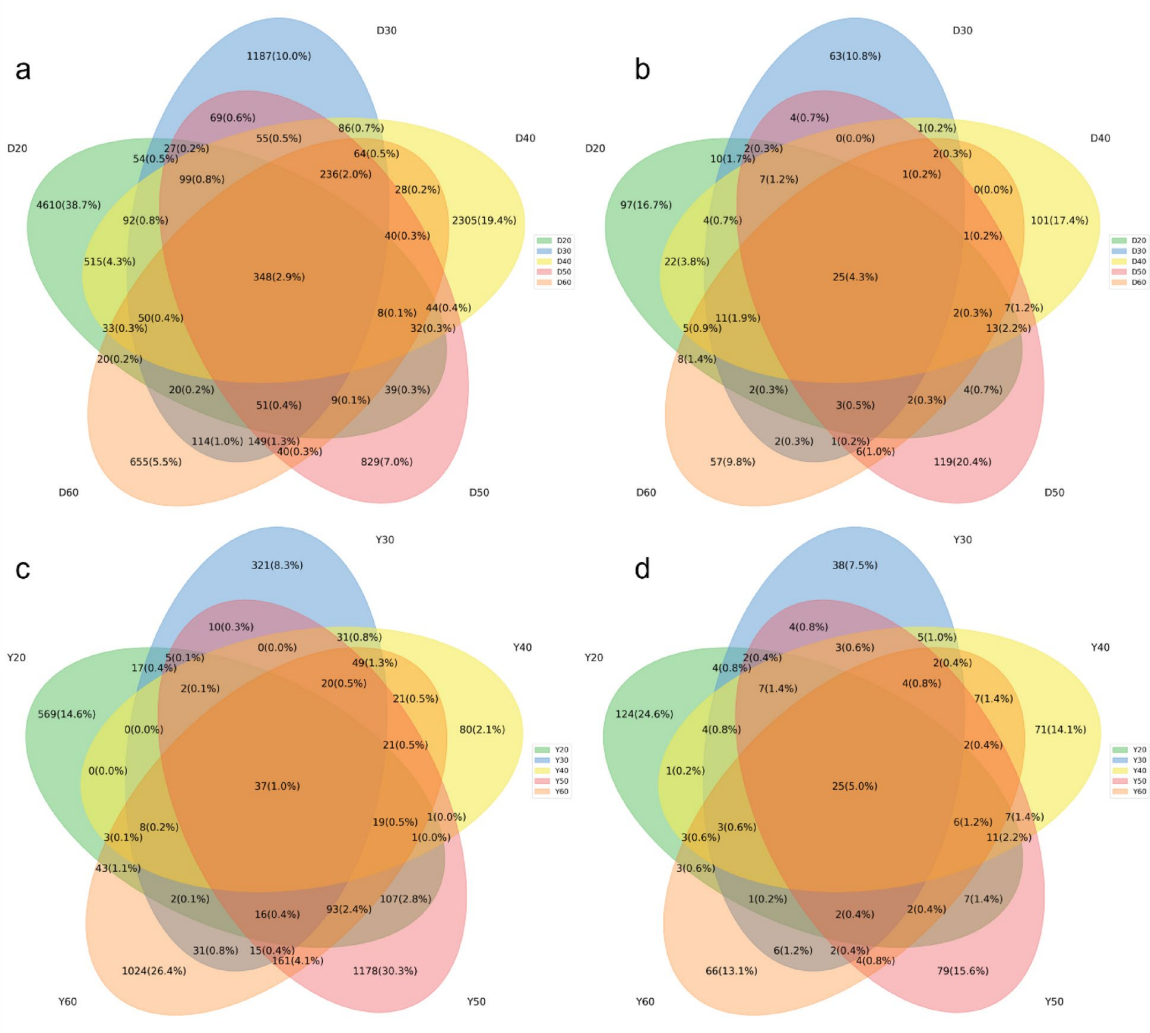
Venn diagrams of shared and unique ASVs in CTLs from Dominica and Yunnan at different fermentation temperatures. **a** Bacterial ASVs in Dominica CTLs; **b** fungal ASVs in Dominica CTLs; **c** bacterial ASVs in Yunnan CTLs; **d** fungal ASVs in Yunnan CTLs

Venn diagrams (Fig. 4) showed that shared ASVs across temperatures accounted for < 5% in both origins, with PERMANOVA pairwise comparisons indicating significant compositional shifts (*p* < 0.01, Cohen’s d = 1.82). Dominica CTLs harbored more unique bacterial ASVs at low temperatures (38.7% at 20 °C), supporting diverse mesophilic metabolism. Yunnan CTLs had more unique bacterial ASVs at 50–60 °C, consistent with thermophilic specialization.

**Fig. 5:**
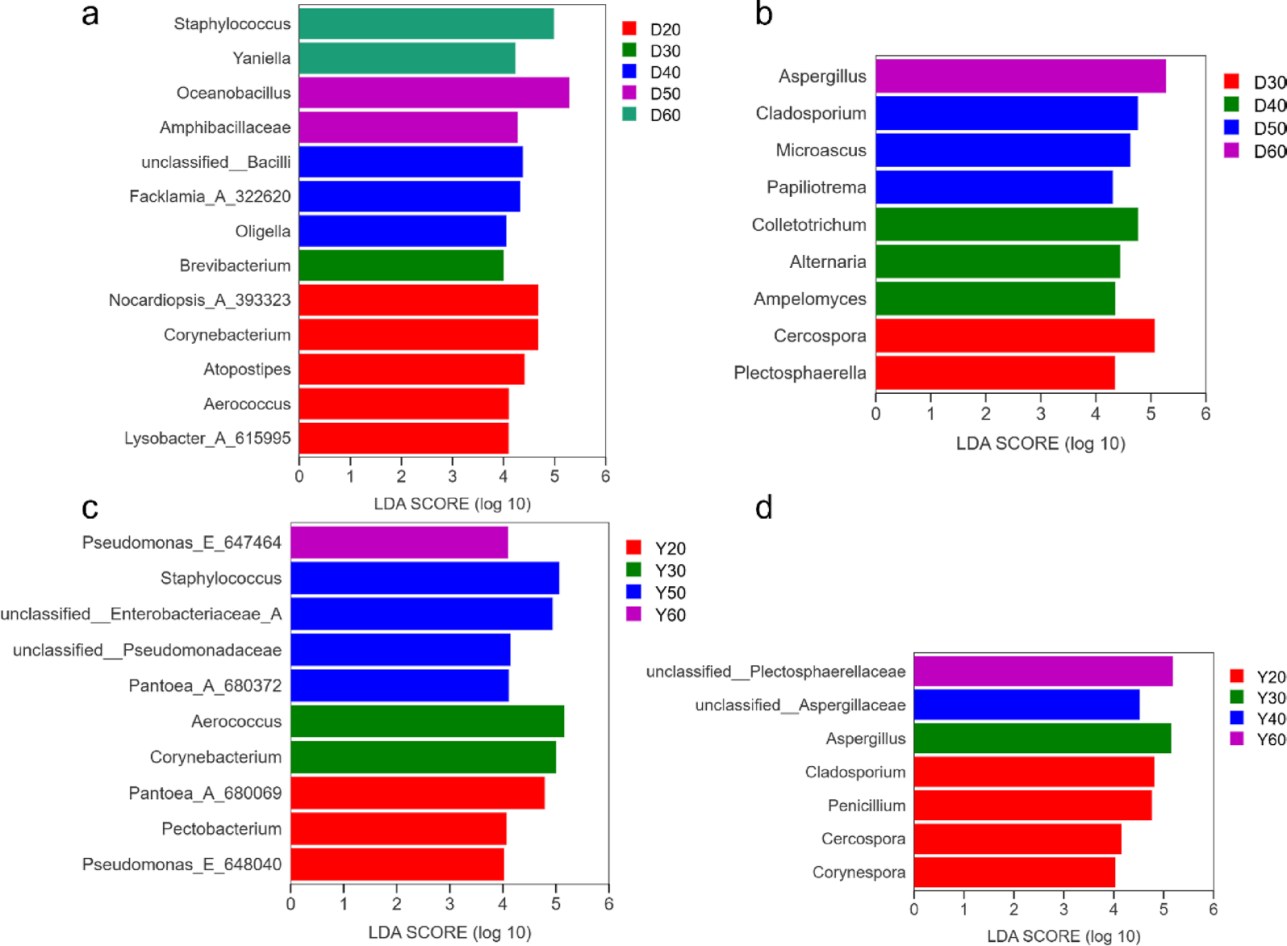
LEfSe analysis of temperature-sensitive microbial biomarkers in CTLs. Bacterial (**a**) and fungal (**b**) biomarkers in Dominica CTLs; bacterial (**c**) and fungal (**d**) biomarkers in Yunnan CTLs. LDA score > 4.0, *p* < 0.05

LEfSe analysis (Fig. 5) identified temperature-sensitive microbial biomarkers (LDA > 4.0, *p* < 0.05). In Dominica CTLs, 22 biomarkers were identified, including mesophilic taxa (*Atopostipes*, *Aerococcus*) at 20 °C, thermophilic taxa (*Oceanobacillus*, *Amphibacillaceae*) at 50 °C, and heat-resistant taxa (*Yaniella*, *Staphylococcus*) at 60 °C. In Yunnan CTLs, 17 biomarkers were identified, with *Staphylococcus* as a key biomarker at 50 °C and *Pseudomonas* at 60 °C. These biomarkers may serve as indicators for monitoring fermentation progression (Wei et al. 2023).

### Changes in volatile flavor compounds with increasing temperature

**Fig. 6:**
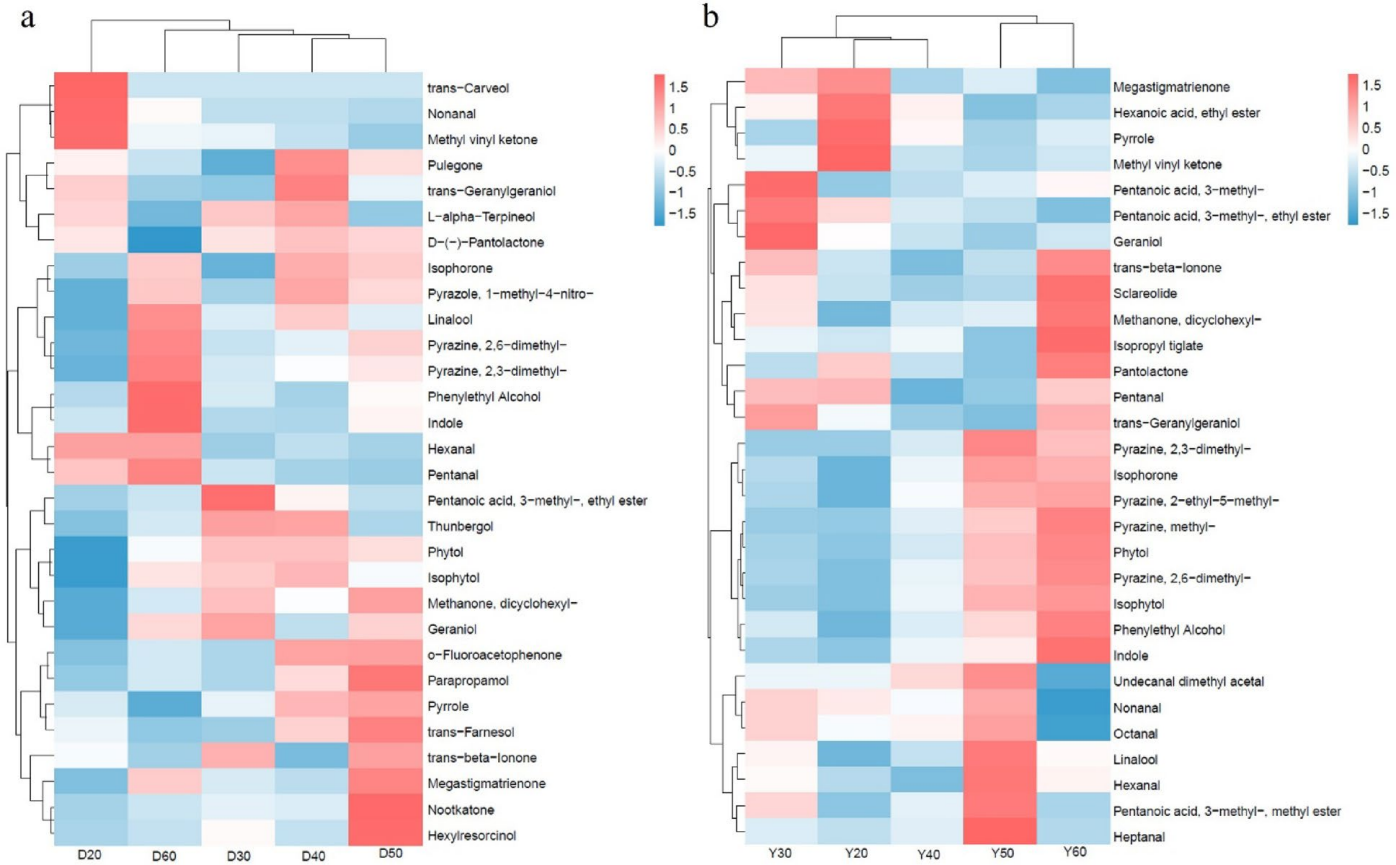
Heatmap of top 30 VFCs in Dominica (**a**) and Yunnan (**b**) CTLs at different fermentation temperatures. Color intensity represents relative content (log10 transformed)

**Fig. 7:**
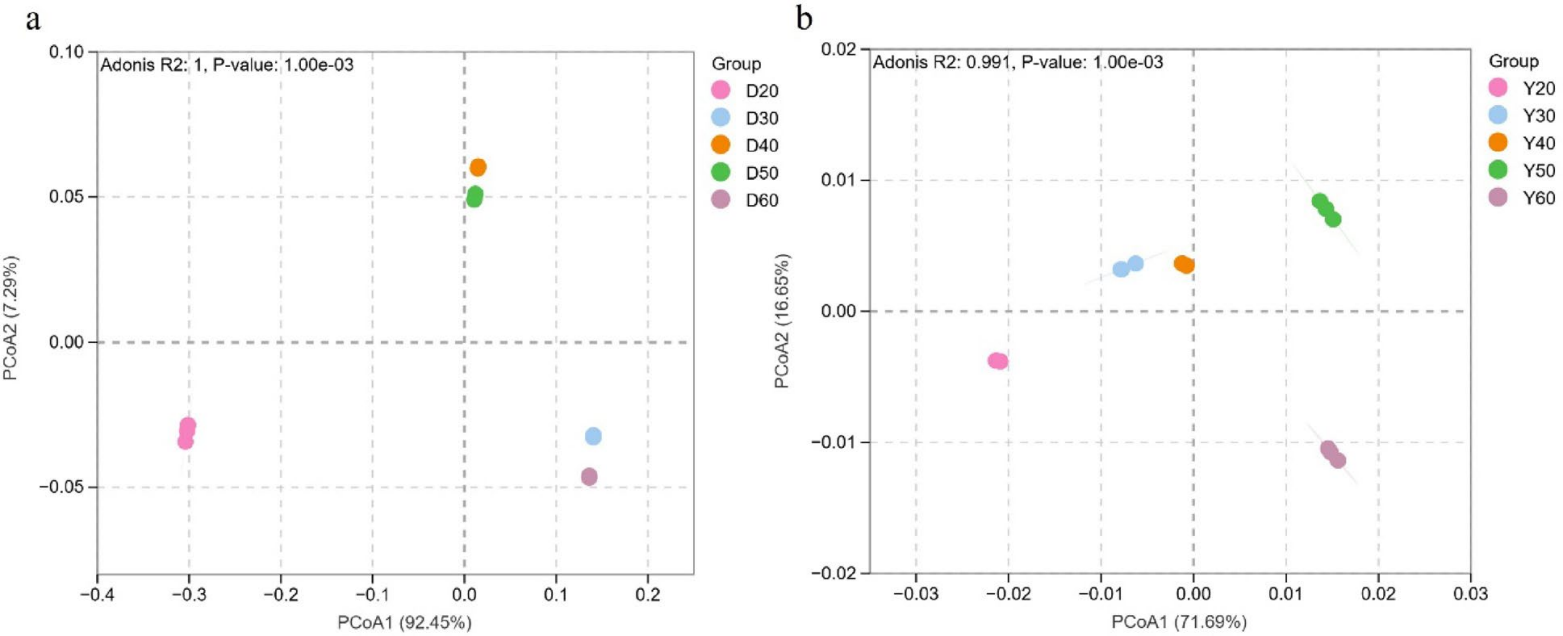
PCA of VFCs in Dominica (**a**) and Yunnan (**b**) CTLs at different fermentation temperatures

A total of 216 VFCs were detected across both origins. The top 30 VFCs by relative abundance were grouped by aroma category for analysis (Fig. 6). PCoA showed clear separation of VFC profiles by temperature and origin (Fig. 7). In Dominica CTLs, VFCs accumulated across multiple temperature stages. At 20 °C, trans-carveol, nonanal, and methyl vinyl ketone predominated, contributing fresh and fruity notes. At 30 °C, esters (3-methyl-pentanoic acid ethyl ester) and alcohols (thunbergol, phytol) increased significantly (1.8–2.5fold ± SD, *p* < 0.01), associated with floral and woody aromas. At 40 °C, pulegone and l-alpha-terpineol increased (2.1–3.0fold ± SD, *p* < 0.01), enhancing spicy and floral notes. At 50 °C, nootkatone increased 4.2fold ± SD (*p* < 0.001), a key contributor to woody and sweet aged tobacco aroma (Leonhardt et al. 2015). At 60 °C, linalool, 2,6-dimethyl-pyrazine, and phenylethyl alcohol increased (1.5–2.2fold ± SD, *p* < 0.05), adding nutty and floral notes.

In contrast, Yunnan CTLs accumulated more VFCs at high temperatures (≥ 50 °C). At 20–40 °C, most VFCs remained low with no significant differences. At 50 °C, undecanal dimethyl acetal, linalool, 2,3-dimethyl-pyrazine, and isophorone increased significantly (3.5–5.0fold ± SD, *p* < 0.001). At 60 °C, trans-beta-ionone, sclareolide, phytol, and indole increased further (indole 5.8fold ± SD, *p* < 0.001). Pyrazines and indole are critical for nutty and roasted aromas (Liu et al. 2024; Mahmoud et al. 2022), suggesting that high temperatures are necessary for characteristic aroma formation in Yunnan CTLs.

Key VFCs showed differential contributions from microbial metabolism versus plant matrix reactions. Pyrazines and indole were primarily derived from microbial metabolism, while nootkatone and ionones resulted from combined microbial and plant contributions. Nootkatone is mainly synthesized by fungi (e.g., *Aspergillus*) via terpene pathways (Leonhardt et al. 2015). Pyrazines are typical microbial metabolites produced by bacteria (e.g., *Staphylococcus*) via Maillard reactions and amino acid decarboxylation (Liu et al. 2024). Indole is synthesized by bacteria through tryptophan metabolism (Mahmoud et al. 2022). Trans-beta-Ionone is formed from carotenoid cleavage, potentially enhanced by microbial esterase activity (Lalko et al. 2007).

### Dynamic changes in microbial interactions with increasing temperature

**Fig. 8:**
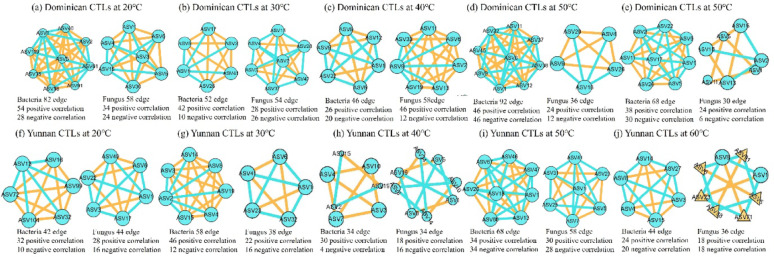
Correlation networks of microbial ASVs (top 10) in Dominica and Yunnan CTLs at different fermentation temperatures. **a**–**e** Dominica CTLs at 20 °C, 30 °C, 40 °C, 50 °C, 60 °C (left: bacteria, right: fungi); **f**–**j** Yunnan CTLs at 20 °C, 30 °C, 40 °C, 50 °C, 60 °C (left: bacteria, right: fungi). Nodes represent ASVs, edges represent correlations (orange: positive, cyan: negative)

Co-occurrence networks of dominant genera (top 30) were constructed based on Spearman correlations (|r|> 0.8, *p* < 0.05, FDR < 0.05, Fig. 8). Topological metrics were calculated (Table 1). These metrics provide insights into community organization and stability. Average degree reflects connectivity: higher values indicate tighter ecological coupling. Modularity (> 0.4 indicates functional specialization, where taxa within the same module share similar ecological niches). Network density represents interaction intensity; denser networks may indicate stronger biotic interactions but also greater susceptibility to perturbation propagation. Network density values exceeding 1 in several samples (e.g., Dominican Bacteria 50 °C: D = 1.051) reflect multiple edges between the same node pairs. In Dominica CTLs, bacterial network complexity was highest at 20 °C (average degree 12.61, modularity 0.63), with positive correlations accounting for 50–80%, suggesting a balanced community structure where cooperation and competition coexist, potentially conferring ecological stability. The number of edges decreased from 84 at 20 °C to 46 at 40 °C, then increased to 82 at 50 °C. Fungal network edges remained stable at low temperatures but decreased significantly above 50 °C, indicating that high temperatures weaken fungal co-occurrence. In Yunnan CTLs, bacterial edges increased from 42 at 20 °C to 68 at 50 °C (average degree 11.33, modularity 0.60). Positive correlations accounted for 50–88%, higher than in Dominica CTLs, indicating that thermal stress may enforce metabolic cooperation among thermophiles-consistent with the stress-gradient hypothesis, where positive interactions become more prevalent under harsh conditions (Kang et al. 2022). However, the proportion of negative correlations increased with temperature, suggesting intensified competitive interactions. Fungal networks maintained stable connectivity (34–58 edges) across temperatures, with positive correlations consistently > 50%.

**Table 1:** Topological metrics of microbial correlation networks

Origin	Microbe	Temperature (°C)	Average degree	Network density	Modularity	Avg clustering coefficient	Avg path length
Dominican	Bacteria	20	12.61	1.077	0.63	0.65	1.9
Dominican	Bacteria	30	9.45	0.945	0.66	0.57	2.1
Dominican	Bacteria	40	9.2	1.022	0.61	0.61	2
Dominican	Bacteria	50	14.15	1.051	0.61	0.63	2
Dominican	Bacteria	60	11.33	1.03	0.61	0.62	2
Dominican	Fungus	20	10.55	1.055	0.62	0.63	1.9
Dominican	Fungus	30	9.82	0.982	0.6	0.59	2
Dominican	Fungus	40	10.55	1.055	0.66	0.63	1.9
Dominican	Fungus	50	8	1	0.63	0.6	2
Dominican	Fungus	60	7.5	1.071	0.66	0.64	1.9
Yunnan	Bacteria	20	8.4	0.933	0.65	0.56	2.1
Yunnan	Bacteria	30	10.55	1.055	0.66	0.63	1.9
Yunnan	Bacteria	40	7.56	0.944	0.68	0.57	2.1
Yunnan	Bacteria	50	11.33	1.03	0.6	0.62	2
Yunnan	Bacteria	60	8.8	0.978	0.61	0.59	2
Yunnan	Fungus	20	8.8	0.978	0.63	0.59	2
Yunnan	Fungus	30	8.44	1.056	0.62	0.63	1.9
Yunnan	Fungus	40	7.56	0.944	0.61	0.57	2.1
Yunnan	Fungus	50	10.55	1.055	0.6	0.63	1.9
Yunnan	Fungus	60	8	1	0.6	0.6	2

These differences align with community structures. Dominica CTLs, with higher microbial diversity, formed complex mixed positive/negative networks, the highest modularity at 20 °C (0.63) and 40 °C (0.66) coincided with peak diversity of aroma-related VFCs (esters, alcohols, ketones), suggesting that modular organization facilitates functional specialization and enhances metabolic output. The decline in fungal network complexity above 50 °C paralleled the accumulation of heat-stable VFCs (pyrazines, indole), indicating a shift from broad metabolic versatility to specialized thermophilic functions. In Yunnan CTLs, the increase in network connectivity at 50 °C (average degree 11.33) coincided with the sharp rise in pyrazines and terpenoids, suggesting that tighter microbial associations under thermal stress promote characteristic high-temperature aroma formation. These patterns imply that network structure mediates the translation of microbial community composition to fermentation functionality. However, co-occurrence networks based on correlation cannot distinguish direct from indirect interactions and are influenced by compositional data bias (Oña et al. 2025). Thus, these patterns provide preliminary insights requiring experimental validation (Srinivasan et al. 2024).

### Relationship between microorganisms and volatile flavor compounds

**Fig. 9:**
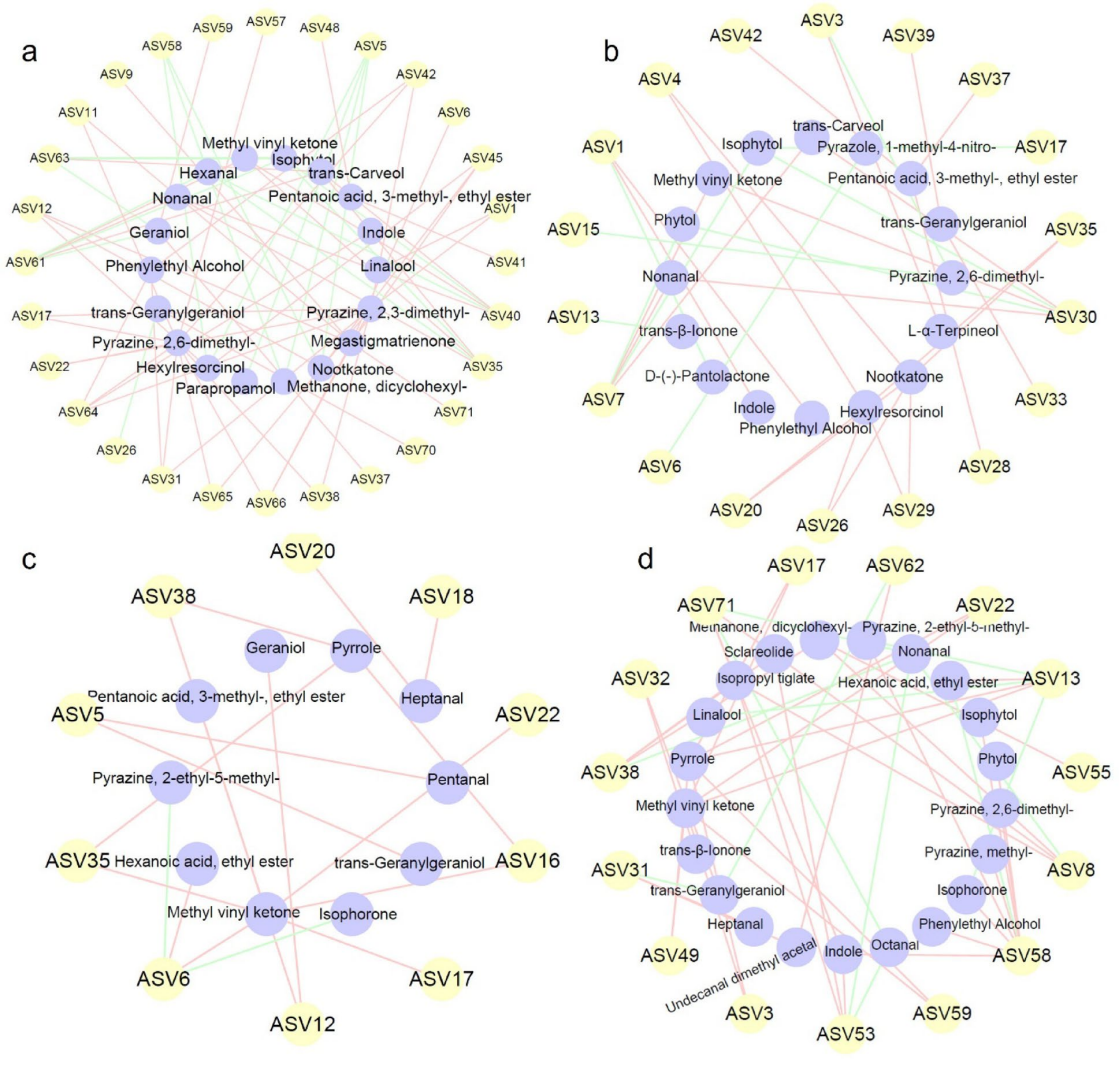
Correlation analysis between microbial ASVs and key VFCs in CTLs. Correlation networks between bacteria (**a**), fungi (**b**) and key VFCs in Dominica CTLs; between bacteria (**c**), fungi (**d**) and key VFCs in Yunnan CTLs. Edges represent significant correlations (|r|> 0.8, *p* < 0.05; red: positive, green: negative)

Correlation analysis between core genera (top 30) and key VFCs (top 30) was performed (Fig. 9). In Dominica CTLs, *Staphylococcus* was positively correlated with pyrazines, indole, and phenylethyl alcohol-compounds associated with nutty, floral, and woody aromas. *Staphylococcus* is known to produce pyrazines via the Maillard reaction and amino acid decarboxylation pathways (Liu et al. 2024), and synthesizes indole through tryptophan metabolism (Mahmoud et al. 2022). *Oceanobacillus* correlated positively with nootkatone, geraniol, and pyrazines, suggesting its potential role in characteristic aroma synthesis. *Oceanobacillus* possesses terpene cyclase genes and has been reported to produce terpenoid compounds under thermal stress (Vala et al. 2025). *Aspergillus* correlated positively with nootkatone, phenylethyl alcohol, and nonanal. *Aspergillus* species are well-documented producers of nootkatone via sesquiterpene biosynthesis pathways, and are known to synthesize phenylethyl alcohol through the shikimate pathway (Wollenberg et al. 2019). *Corynebacterium* showed negative correlations with several VFCs, possibly due to indirect interactions, metabolic competition, or inhibitory effects. In Yunnan CTLs, *Staphylococcus* correlated positively with heptanal and methyl vinyl ketone. These short-chain aldehydes and ketones are common products of fatty acid metabolism and amino acid catabolism, processes in which *Staphylococcus* is actively involved (Nazipi et al. 2021). *Oceanobacillus* correlated positively with pentanal and trans-geranylgeraniol. Geranylgeraniol is a diterpenoid precursor; the correlation with *Oceanobacillus* aligns with its thermophilic lifestyle and capacity for terpenoid synthesis under heat stress (Gutbrod et al. 2019). *Aspergillus* correlated with 13 VFCs, including phenylethyl alcohol, indole, and pyrazines. This broad correlation is consistent with the metabolic versatility of *Aspergillus*, which possesses extensive secondary metabolite gene clusters involved in the biosynthesis of diverse aroma compounds (Wollenberg et al. 2019).

Temperature-sensitive biomarkers identified by LEfSe showed strong correlations with VFCs. For instance, *Staphylococcus* correlated with pyrazines and indole; *Oceanobacillus* with nootkatone; *Aspergillus* with nootkatone and phenylethyl alcohol. These results suggest that temperature drives the succession of microbial biomarkers potentially associated with VFC accumulation, forming a conceptual “temperature–microorganism-VFC” framework. However, all observed relationships are correlational; no direct experimental evidence (e.g., pure culture fermentation) was provided to confirm biosynthetic roles. Functional validation is essential to establish causality. While our analysis focused on the top 30 ASVs, low-abundance taxa may also contribute to VFC formation (Lynch et al. 2015). Rare taxa can serve as a functional reservoir, harboring metabolic capabilities that become activated under specific conditions (Jousset et al. 2017). Conditionally rare taxa disproportionately contribute to community functions when environmental conditions favor their growth (Shade et al. 2014). Moreover, keystone species in microbial networks are not always abundant, yet their removal can destabilize community function (Banerjee et al. 2018). In our LEfSe analysis, several biomarkers with LDA > 4.0 (e.g., *Atopostipes*, *Amphibacillaceae*) were not among the top 30 ASVs, yet their temperature-specific enrichment suggests functional relevance. Future studies employing deeper sequencing, metagenomic assembly, or targeted isolation are needed to capture the functional potential of these rare taxa and establish causal links to aroma formation.

## Conclusions

This study systematically explored temperature-associated microbial succession and its correlation with VFC formation during CTL fermentation under a 20–60 °C continuous gradient. Temperature significantly reshapes microbial community structure, with distinct succession patterns between origins. Dominica CTLs exhibit more diverse and evenly distributed communities, while Yunnan CTLs are dominated by thermotolerant bacteria (e.g., *Staphylococcus*). A total of 216 VFCs were detected, with origin-specific formation patterns: Dominica CTLs produce abundant esters, alcohols, and ketones across temperature stages, whereas Yunnan CTLs accumulate more pyrazines, indole, and terpenoids at high temperatures (≥ 50 °C). Co-occurrence patterns differ: Dominica CTLs form complex mixed networks; Yunnan CTLs develop stable positive co-occurrence consortia at high temperatures. Temperature-driven microbial succession is correlated with VFC accumulation, forming a conceptual “temperature-microorganism-VFC” framework.

Temperature-sensitive biomarkers identified in this study represent candidate strains for microbial inoculant development, pending functional validation. Based on correlational evidence, hypotheses for industrial optimization (requiring sensory and pilot-scale validation) include: fermenting Dominica CTLs within 40–50 °C to balance microbial diversity and VFC accumulation, and fermenting Yunnan CTLs at 50–60 °C to favor thermotolerant microbes and characteristic aroma enrichment. These recommendations are speculative without direct functional validation; future studies should focus on strain isolation, co-culture experiments, enzyme activity assays, and pilot-scale trials to confirm causality and scalability.

## Abbreviations

CTLs: Cigar tobacco leaves
VFCs: Volatile flavor compounds
LEfSe: Linear discriminant analysis effect size
HS-SPME-GC-MS: Headspace solid-phase microextraction coupled to gas chromatography-mass spectrometry
ANOVA: One-way analysis of variance
ASVs: Amplicon sequence variants
PCoA: Principal coordinate analysis
LDA: Linear discriminant analysis
CLR: Centered log-ratio
FDR: False discovery rate

## References

[CR1] Banerjee S, Schlaeppi K, van der Heijden MGA (2018) Keystone taxa as drivers of microbiome structure and functioning. Nat Rev Microbiol 16:567–576. 10.1038/s41579-018-0024-1 29789680 10.1038/s41579-018-0024-1

[CR2] Bolyen E, Rideout JR, Dillon MR et al (2019) Reproducible, interactive, scalable and extensible microbiome data science using QIIME 2. Nat Biotechnol 37:852–857. 10.1038/s41587-019-0209-9 31341288 10.1038/s41587-019-0209-9 PMC7015180

[CR3] Callahan BJ, McMurdie PJ, Rosen MJ et al (2016) DADA2: high-resolution sample inference from Illumina amplicon data. Nat Methods 13:581–583. 10.1038/nmeth.3869 27214047 10.1038/nmeth.3869 PMC4927377

[CR4] Cai W, Zhang Q, Zhu P et al (2025) Study on dynamic changes and correlations of microbial diversity, key enzyme activity and conventional components in tobacco leaves during the aging process. Bioresour Bioprocess 12:130. 10.1186/s40643-025-00969-3 41191230 10.1186/s40643-025-00969-3 PMC12589753

[CR5] Egbune EO, Ezedom T, Odeghe OB et al (2023) Solid-state fermentation production of L-lysine by *Corynebacterium glutamicum* (ATCC 13032) using agricultural by-products as substrate. World J Microbiol Biotechnol 40:20. 10.1007/s11274-023-03822-x 37996724 10.1007/s11274-023-03822-x

[CR6] Gao Y, Wang Y, Hou B et al (2025) Diversity of microbial communities in cigar filler leaves with different initial water contents analyzed based on high-throughput sequencing technology. Front Microbiol 16:1508866. 10.3389/fmicb.2025.1508866 39990154 10.3389/fmicb.2025.1508866 PMC11845121

[CR7] Geib E, Baldeweg F, Doerfer M et al (2019) Cross-chemistry leads to product diversity from Atromentin synthetases in *Aspergilli* from section Nigri. Cell Chem Biol 26:223-234.e6. 10.1016/j.chembiol.2018.10.021 30527997 10.1016/j.chembiol.2018.10.021

[CR8] Gutbrod K, Romer J, Dörmann P (2019) Phytol metabolism in plants. Prog Lipid Res 74:1–17. 10.1016/j.plipres.2019.01.002 30629961 10.1016/j.plipres.2019.01.002

[CR9] Han X, Ma T, Wu Y et al (2025) Assessment of wheat Qu fermented at medium and high temperatures: effects of Bupleurum addition on fermentation characteristics, volatile profiles, and microbial communities. Food Res Int 203:115814. 10.1016/j.foodres.2025.115814 40022341 10.1016/j.foodres.2025.115814

[CR10] Han P, Guo D, Zhang M et al (2026) Integrated multi-omics reveals microbial and metabolic mechanisms driving enhanced fermentation quality in cigar tobacco leaves with exogenous additives. Bioresour Bioprocess 13:2. 10.1186/s40643-025-00998-y 41489776 10.1186/s40643-025-00998-y PMC12770121

[CR11] Jia Y, Guo S, Hu W et al (2025) Effects of different fermentation temperatures on microbiomes of cigar tobacco leaves. Front Bioeng Biotechnol 13:1550383. 10.3389/fbioe.2025.1550383 40070551 10.3389/fbioe.2025.1550383 PMC11893599

[CR12] Jousset A, Bienhold C, Chatzinotas A et al (2017) Where less may be more: how the rare biosphere pulls ecosystems strings. ISME J 11:853–862. 10.1038/ismej.2016.174 28072420 10.1038/ismej.2016.174 PMC5364357

[CR13] Kind T, Wohlgemuth G, Lee DY et al (2009) FiehnLib: mass spectral and retention index libraries for metabolomics based on quadrupole and time-of-flight gas chromatography/mass spectrometry. Anal Chem 81:10038–10048. 10.1021/ac9019522 19928838 10.1021/ac9019522 PMC2805091

[CR14] Knapp BD, Willis L, Gonzalez C et al (2025) Metabolic rearrangement enables adaptation of microbial growth rate to temperature shifts. Nat Microbiol 10:185–201. 10.1038/s41564-024-01841-4 39672961 10.1038/s41564-024-01841-4

[CR15] Lalko J, Lapczynski A, McGinty D et al (2007) Fragrance material review on trans-beta-ionone. Food Chem Toxicol 45(Suppl 1):S248–S250. 10.1016/j.fct.2007.09.011 18031901 10.1016/j.fct.2007.09.011

[CR16] Leonhardt RH, Berger RG (2015) Nootkatone. Adv Biochem Eng Biotechnol 148:391–404. 10.1007/10_2014_279 25326849 10.1007/10_2014_279

[CR17] Liu X, Quan W (2024) Progress on the synthesis pathways and pharmacological effects of naturally occurring pyrazines. Molecules 29:3597. 10.3390/molecules29153597 39125002 10.3390/molecules29153597 PMC11314619

[CR18] Lynch MDJ, Neufeld JD (2015) Ecology and exploration of the rare biosphere. Nat Rev Microbiol 13:217–229. 10.1038/nrmicro3400 25730701 10.1038/nrmicro3400

[CR19] Ma L, Wang Y, Wang X et al (2024) Solid-state fermentation improves tobacco leaves quality via the screened *Bacillus subtilis* of simultaneously degrading starch and protein ability. Appl Biochem Biotechnol 196:506–521. 10.1007/s12010-023-04486-x 37148443 10.1007/s12010-023-04486-x

[CR20] Mahmoud E, Hayallah AM, Kovacic S et al (2022) Recent progress in biologically active indole hybrids: a mini review. Pharmacol Rep 74:570–582. 10.1007/s43440-022-00370-3 35594012 10.1007/s43440-022-00370-3

[CR21] Nazipi S, Vangkilde-Pedersen SG, Busck MM et al (2021) An antimicrobial *Staphylococcus sciuri* with broad temperature and salt spectrum isolated from the surface of the African social spider, *Stegodyphus dumicola*. Antonie Van Leeuwenhoek 114:325–335. 10.1007/s10482-021-01526-6 33543432 10.1007/s10482-021-01526-6

[CR22] Omaiye EE, Luo W, McWhirter KJ, Pankow JF et al (2022) Flavour chemicals, synthetic coolants and pulegone in popular mint-flavoured and menthol-flavoured e-cigarettes. Tob Control 31:e3–e9. 10.1136/tobaccocontrol-2021-056582 34193607 10.1136/tobaccocontrol-2021-056582 PMC8716610

[CR23] Oña L, Shreekar SK, Kost C (2025) Disentangling microbial interaction networks. Trends Microbiol 33:619–634. 10.1016/j.tim.2025.01.013 40044528 10.1016/j.tim.2025.01.013

[CR24] Qu FF, Li XH, Wang PQ et al (2023) Effect of thermal process on the key aroma components of green tea with chestnut-like aroma. J Sci Food Agric 103:657–665. 10.1002/jsfa.12177 36054006 10.1002/jsfa.12177

[CR25] Ren M, Qin Y, Zhang L et al (2023) Effects of fermentation chamber temperature on microbes and quality of cigar wrapper tobacco leaves. Appl Microbiol Biotechnol 107:6469–6485. 10.1007/s00253-023-12750-7 37665370 10.1007/s00253-023-12750-7

[CR26] Saishu N, Morimoto K, Yamasato H et al (2015) Characterization of *Aerococcus viridans* isolated from milk samples from cows with mastitis and manure samples. J Vet Med Sci 77:1037–1042. 10.1292/jvms.15-0100 25843745 10.1292/jvms.15-0100 PMC4591142

[CR27] Shade A, Jones SE, Caporaso JG et al (2014) Conditionally rare taxa disproportionately contribute to temporal changes in microbial diversity. Mbio 5:e01371–e01371. 10.1128/mBio.01371-14 25028427 10.1128/mBio.01371-14 PMC4161262

[CR28] Shan XJ, Jin L, Li F et al (2025) Isolation of indigenous *Bacillus velezensis* from aging tobacco leaves for improving the flavor of flue-cured tobacco. Front Microbiol 16:1623279. 10.3389/fmicb.2025.1623279 40740331 10.3389/fmicb.2025.1623279 PMC12307346

[CR29] Song B, Li Y, Yu Z, Jin J et al (2024) Changes in enzyme activity, structure and growth strategies of the rhizosphere microbiome influenced by elevated temperature and CO₂. Sci Total Environ 954:176522. 10.1016/j.scitotenv.2024.176522 39326750 10.1016/j.scitotenv.2024.176522

[CR30] Srinivasan S, Jnana A, Murali TS (2024) Modeling microbial community networks: methods and tools for studying microbial interactions. Microb Ecol 87:56. 10.1007/s00248-024-02370-7 38587642 10.1007/s00248-024-02370-7 PMC11001700

[CR31] Takahashi Y, Horiyama S, Honda C et al (2013) A chemical approach to searching for bioactive ingredients in cigarette smoke. Chem Pharm Bull (Tokyo) 61:85–89. 10.1248/cpb.c12-00539 23302590 10.1248/cpb.c12-00539

[CR32] Vala V, Suhagia T, Raina V et al (2025) Thermostable amylases from thermophilic microbes: advances in production, engineering, and industrial applications. Nanotechnology. 10.1088/1361-6528/ae2f66 41418326 10.1088/1361-6528/ae2f66

[CR33] Wei J, Song K, Zang Z et al (2024) Influence of specific tobacco endophytic *Bacillus* on tobacco leaf quality enhancement during fermentation. Front Microbiol 15:1468492. 10.3389/fmicb.2024.1468492 39654681 10.3389/fmicb.2024.1468492 PMC11625772

[CR34] Wei W, Li Y, Huang T (2023) Using machine learning methods to study colorectal cancer tumor micro-environment and its biomarkers. Int J Mol Sci 24:11133. 10.3390/ijms241311133 37446311 10.3390/ijms241311133 PMC10342679

[CR35] Wieder C, Simon-Sánchez C, Liermann J et al (2025) Allantofuranone biosynthesis and precursor-directed mutasynthesis of hydroxylated analogues. J Nat Prod 88:1191–1200. 10.1021/acs.jnatprod.5c00197 40247749 10.1021/acs.jnatprod.5c00197 PMC12105029

[CR36] Wu X, Hu Y, Wang Q et al (2023) Study on the correlation between the dominant microflora and the main flavor substances in the fermentation process of cigar tobacco leaves. Front Microbiol 14:1267447. 10.3389/fmicb.2023.1267447 38075898 10.3389/fmicb.2023.1267447 PMC10699171

[CR37] Wu Q, Duan X, Tang D et al (2025) Foliar microbiota confers deeper color of fermented cigar wrapper under additional fermented bacteria. Bioresour Bioprocess 12:78. 10.1186/s40643-025-00921-5 40694219 10.1186/s40643-025-00921-5 PMC12283526

[CR38] Yang D, Kato H, Kawatsu K et al (2022) Reconstruction of a soil microbial network induced by stress temperature. Microbiol Spectr 10:e0274822. 10.1128/spectrum.02748-22 35972265 10.1128/spectrum.02748-22 PMC9602341

[CR39] Yang L, Fan W, Xu Y (2024) Effects of storage period and season on the microecological characteristics of high-temperature Daqu. Food Res Int 196:115034. 10.1016/j.foodres.2024.115034 39614477 10.1016/j.foodres.2024.115034

[CR40] Yu C, Li M, Zhang B et al (2022) Hydrothermal pretreatment contributes to accelerate maturity during the composting of lignocellulosic solid wastes. Bioresour Technol 346:126587. 10.1016/j.biortech.2021.126587 34933104 10.1016/j.biortech.2021.126587

[CR41] Yu Y, Yang Y, Jia T et al (2025) Changes in microbial composition during flue-cured tobacco aging and their effects on chemical composition: a review. Bioresour Bioprocess 12:71. 10.1186/s40643-025-00883-8 40397057 10.1186/s40643-025-00883-8 PMC12095124

[CR42] Zhang G, He Y, Yang W et al (2025) Integrated microbiology and metabolomics analysis reveal the fermentation process and the flavor development in cigar tobacco leaf. Microbiol Spectr 13:e0102924. 10.1128/spectrum.01029-24 40272187 10.1128/spectrum.01029-24 PMC12131730

[CR43] Zhang G, Zhao L, Li W et al (2023a) Changes in physicochemical properties and microbial community succession during leaf stacking fermentation. AMB Express 13:132. 10.1186/s13568-023-01642-8 37991629 10.1186/s13568-023-01642-8 PMC10665287

[CR44] Zhang Q, Huang Y, An H et al (2024a) The impact of gradient variable temperature fermentation on the quality of cigar tobacco leaves. Front Microbiol 15:1433656. 10.3389/fmicb.2024.1433656 39735193 10.3389/fmicb.2024.1433656 PMC11672604

[CR45] Zhang Q, Kong G, Zhao G et al (2023b) Microbial and enzymatic changes in cigar tobacco leaves during air-curing and fermentation. Appl Microbiol Biotechnol 107:5789–5801. 10.1007/s00253-023-12663-5 37458766 10.1007/s00253-023-12663-5 PMC10439857

[CR46] Zhang Y, Lin B, Hao Y et al (2024b) Two-stage inoculation with lignocellulose-degrading microorganisms in composting: enhanced humification efficiency and underlying mechanisms. Environ Res 271:120906. 10.1016/j.envres.2025.120906 10.1016/j.envres.2025.120906 39947380

[CR47] Zheng T, Zhang Q, Li P et al (2022) Analysis of microbial community, volatile flavor compounds, and flavor of cigar tobacco leaves from different regions. Front Microbiol 13:907270. 10.3389/fmicb.2022.907270 35756070 10.3389/fmicb.2022.907270 PMC9231593

[CR48] Zhong R, Sun Z, Feng L et al (2025) Decoding honey-sweet flavored flue-cured tobacco from Guizhou with data science and flavoromics by volatile and cell wall components. Front Chem 13:1613828. 10.3389/fchem.2025.1613828 41103910 10.3389/fchem.2025.1613828 PMC12521107

